# Simultaneous multiplex genome loci editing of *Halomonas bluephagenesis* using an engineered CRISPR-guided base editor

**DOI:** 10.1016/j.synbio.2024.04.016

**Published:** 2024-04-25

**Authors:** Yulin Zhang, Yang Zheng, Qiwen Hu, Zhen Hu, Jiyuan Sun, Ping Cheng, Xiancai Rao, Xiao-Ran Jiang

**Affiliations:** a Medical Research Institute, Southwest University, Chongqing, 400716, China; b Department of Microbiology, College of Basic Medical Sciences, Army Medical University (Third Military Medical University), Chongqing, 400038, China

**Keywords:** Base editor, Gene editing, Multiplex, *Halomonas bluephagenesis*

## Abstract

*Halomonas bluephagenesis* TD serves as an exceptional chassis for next generation industrial biotechnology to produce various products. However, the simultaneous editing of multiple loci in *H. bluephagenesis* TD remains a significant challenge. Herein, we report the development of a multiple loci genome editing system, named CRISPR-deaminase-assisted base editor (CRISPR-BE) in *H. bluephagenesis* TD. This system comprises two components: a cytidine (CRISPR-cBE) and an adenosine (CRISPR-aBE) deaminase-based base editor. CRISPR-cBE can introduce a cytidine to thymidine mutation with an efficiency of up to 100 % within a 7-nt editing window in *H. bluephagenesis* TD. Similarly, CRISPR-aBE demonstrates an efficiency of up to 100 % in converting adenosine to guanosine mutation within a 7-nt editing window. CRISPR-cBE has been further validated and successfully employed for simultaneous multiplexed editing in *H. bluephagenesis* TD. Our findings reveal that CRISPR-cBE efficiently inactivated all six copies of the IS1086 gene simultaneously by introducing stop codon. This system achieved an editing efficiency of 100 % and 41.67 % in inactivating two genes and three genes, respectively. By substituting the *P*_cas_ promoter with the inducible promoter *P*_Mmp1_, we optimized CRISPR-cBE system and ultimately achieved 100 % editing efficiency in inactivating three genes. In conclusion, our research offers a robust and efficient method for concurrently modifying multiple loci in *H. bluephagenesis* TD, opening up vast possibilities for industrial applications in the future.

## 1 Introduction

Halophilic *Halomonas* species have attracted significant attention from researchers in industrial biotechnology [1]. The unique ability to thrive in high concentrations of NaCl and alkaline pH environments enables *Halomonas* bacteria to facilitate contamination-free fermentation processes under unsterile conditions and support continuous production. The continuous production not only simplifies processes but also leads to substantial cost reductions for numerous commercial products [[2], [3], [4], [5], [6], [7], [8], [9], [10]]. *H. bluephagenesis* strain TD, a halophilic bacterium isolated from Aydingol Lake in Xinjiang Province, China, has emerged as a promising chassis for synthesizing various types of bioplastics polyhydroxyalkanoates (PHAs) [[4], [5], [6], [7], [11], [12]]. *H. bluephagenesis* TD thrives in NaCl concentrations ranging from 20 to 150 g/L, with an optimal range of 40–60 g/L. The strain exhibits growth potential within a pH range of 5.0–11.0, with optimal growth observed at pH levels between 8.5 and 9.0. Pilot-scale PHA production has been successfully conducted in a 5,000 L bioreactor vessel, operating under open unsterile conditions [8]. Furthermore, *H. bluephagenesis* TD has been successfully developed as an outstanding chassis for next-generation industrial biotechnology (NGIB), capable of producing multiple products [1,10,[13], [14], [15]]. These findings demonstrate that *H. bluephagenesis* TD serves as a valuable platform or chassis for further molecular engineering studies.

Efficient genetic manipulation or gene editing methods are crucial for effectively regulating natural metabolic pathways to produce products. While traditional methods for gene knockout in *H. bluephagenesis* TD are already established, they often suffer from inefficiency, time consuming, or labor intensive [2,4]. The CRISPR/Cas9 system has been developed for genome editing in *H. bluephagenesis* TD [16]. However, the requirement of a homologous arm for each editing target in CRISPR/Cas9 genome editing in *H. bluephagenesis* TD poses a challenge when attempting to simultaneously edit multiple loci in a single iteration of the experiment. Additionally, CRISPR/Cas9-induced double-strand breaks (DSBs) can lead to excessive damage to the genome DNA, significantly reducing the efficiency of plasmid transformation [[17], [18], [19], [20]]. Consequently, the simultaneous editing of multiple loci remains a significant challenge that has not yet been achieved in *H. bluephagenesis* TD.

The rise of CRISPR-BE, a novel approach that utilizes CRISPR-guided base editors, presents a promising alternative for traditional CRISPR methodologies and has attracted significant attention in the field of microbial genome editing. These base editors exhibit remarkable efficiency and precision in inducing targeted point mutations in the genome, without the necessity for DSBs. By combining the functionality of cytidine or adenosine deaminase with the precise genome targeting capabilities of various Cas9 variants, the CRISPR-guided cytidine deaminase base editor (CRISPR-cBE) facilitates the conversion of C to T [21,22], while the CRISPR-guided adenosine deaminase base editor (CRISPR-aBE) accomplishes the transformation of A to G [23,24]. Furthermore, CRISPR-cBE mediates the conversion of C to T in specific codons such as CAA (Gln), CAG (Gln), CGA (Arg), and TGG (Trp), generating premature stop codons (TAA, TAG or TGA) [25,26]. The catalytically inactive dead Cas9 (dCas9) or partially inactive nickase Cas9 (nCas9) domains are the most commonly used. By fusing dCas9 with cytidine deaminase, CRISPR/AID editing has been proved to be functional inactivation a single gene in *H. bluephagenesis* TD [27]. Additionally, the nCas9's ability to nick DNA strand stimulates cellular mismatch repair (MMR) [21,28], enhancing the base editing efficiency when used as a delivery system for the deaminases in some microorganisms [25,29,30]. The CRISPR-BE system has been proven effective in genetic engineering and genome-wide gene function identification [31].

Herein, we report the establishment, validation, and multiplexed genome editing of CRISPR-BE in *H. bluephagenesis* TD. CRISPR-BE, comprised of nCas9 fused with cytidine or adenosine deaminase, offers a time saving approach for accurately editing target bases in *H. bluephagenesis* TD with an efficiency of up to 100 %. Furthermore, CRISPR-cBE achieved 100 % editing efficiency in inactivating two genes, and 41.67 % in inactivating three genes. To further enhance the inactivation efficiency of multiple genes, we optimized CRISPR-cBE by substituting the *P*_cas_ promoter with the inducible promoter *P*_Mmp1_, ultimately achieving 100 % editing efficiency in inactivating three genes. CRISPR-cBE's independence from the host's recombinant enzymes or DSB repair pathways positions it as a potentially widespread tool in metabolic engineering and directed evolution in *H. bluephagenesis* TD.

## 2 Methods

### 2.1 Strains and culture conditions

Bacterial strains utilized in this study are listed in Supplementary Table 1. *H. bluephagenesis* strain TD, has been deposited in the China General Microbiological Culture Collection Center (CGMCC) under the collection number 4353. *Escherichia coli* S17-1 served as the host for plasmid construction and as the conjugation donor. The recombinant plasmids were transformed into chemically competent *E. coli* S17-1 through heat-shock and subsequently propagated in Luria–Bertani (LB) broth or on LB agar plates, with the addition of either chloramphenicol (Cm, 25 μg/mL) or kanamycin (Kan, 50 μg/mL) for selection purposes. *H. bluephagenesis* TD and its derivatives were cultivated with 60-LB medium (LB medium supplemented with 60 g/L NaCl) or 60-LB agar at 37 °C. Whenever required, the media were supplemented with Cm (25 μg/mL) or spectinomycin (Spe, 100 μg/mL) for maintaining plasmids.

### 2.2 Plasmid construction

Plasmids used in this study are listed in Supplementary Table 2. Primers used in this study are listed in Supplementary Table 3. Primers were synthesized by BGI Genomics (China). DNA fragments were amplified using PrimeSTAR® Max DNA Polymerase (Takara Bio, Japan). The plasmids were generated using the Gibson Assembly Method [32] with the Gibson Assembly Master Mix Kit (New England Biolabs, USA). Restriction endonuclease BsaI-HF®v2 and T4 DNA ligase used for Golden Gate assembly were purchased from New England Biolabs (USA). Plasmid extraction kits were purchased from Tiangen Biotech Co., Ltd. (China).

The CRISPR-BE plasmid pxBE3 and pABE7.10 were constructed using pSEVA321 as the backbone plasmid. The rAPOBEC1 and uracil glycosylase inhibitor (UGI) were amplified from plasmid pCRISPR-cBEST (Addgene plasmid no. 125689), and *ec*TadA was amplified from plasmid pCRISPR-aBEST (Addgene plasmid no. 131464). The fusion protein, fusing the rAPOBEC1 to the N-terminal of nCas9 (D10A) and the UGI to its C-terminus or fusing the *ec*TadA to the N-terminal of nCas9 (D10A), was controlled by the constitutive *P*_c__as_ promoter. The sgRNAs for gene editing were obtained from CRISPy-web (https://crispy.secondarymetabolites.org). The sgRNA sequences used in this study are given in Supplementary Table 4 and Supplementary Table 5. The vector pSEVA341 was used to construct the sgRNA plasmids, with the sgRNA arrays inserted into the sgRNA scaffolds through Golden Gate assembly. The transcription of the sgRNA was controlled by the constitutive promoter *P*_J23119_ and terminated via a *t0* terminator. Finally, all reaction setups were chemically transformed into *E. coli* S17-1, and verified through DNA sequencing.

### 2.3 Conjugation

*H. bluephagenesis* TD proved resistant to DNA transformation via both electroporation and chemical methods. Consequently, we employed conjugation to introduce the desired plasmid, using *E. coli* S17-1 as the donor strain [33]. Once grown to the logarithmic phase, 1 mL aliquots of both *E. coli* S17-1 donor and *H. bluephagenesis* TD cells were harvested and centrifuged at 2,500×*g* for 2 min. After discarding the supernatant, the bacterial cell pellets were resuspended in 20-LB medium (LB medium supplemented with 20 g/L NaCl) and mixed in a 1:1 ratio. This mixture was then incubated on 20-LB agar plates at 37 °C for 6 h. Subsequently, the mixed bacteria were plated onto plates containing the appropriate antibiotics and incubated at 37 °C for 24–48 h to isolate single colonies. These colonies were then validated through PCR analysis.

### 2.4 *In vivo* spacer-matrix design

As two key components of the sgRNA-matrix are the distinct sequence context and the position of the target nucleotide. The pattern of the CRISPR-cBE sgRNA matrix is N_1-2_(TC_n_GC_n_AC_n_C_n_)N_11-12_NGG, where n = 3 to 9, as opposed to the pattern of the CRISPR-aBE sgRNA matrix, which is N_2-3_(TA_n_GA_n_CA_n_A_n_)N_10-11_NGG, where n = 4 to 10. Theoretically, each matrix should contain in total seven pieces of sgRNA. Sequence pattern as previously described was used to locate all possible sgRNAs in the genome of *H. bluephagenesis* TD [25]. The determination of whether the ones are located within essential genes or not was conducted through a manual examination, utilizing the genome annotation data of *H. bluephagenesis* TD. Seven pieces of sgRNAs for both CRISPR-cBE and CRISPR-aBE were finally selected (Supplementary Table 5).

### 2.5 Validating base pair changes by sanger sequencing

Primers were designed to amplify a multi-hundred base pair region containing the base editing window (Supplementary Table 3). Single colonies grown after conjugation were streaked and purified on fresh agar plates. Colony PCR was performed to amplify the designed regions from *H. bluephagenesis* TD colonies. PCR products purification and Sanger sequencing were conducted by Tsingke Biotechnology Co., Ltd.

For mixed trace signals, the call secondary peak function of CLC Main Workbench 23.0.5 (QIAGEN Bioinformatics) was applied to calculate the editing efficiency. The editing efficiency of the modified site was obtained by calculating the peak ratio of the secondary peaks [25].

### 2.6 Genome editing using CRISPR-BE

The psgRNA plasmid targeting the *phaC* gene was generated and further transformed into *H. bluephagenesis* TD1.0 (A novel T7-like RNA polymerase-integrated derivative of TD, Supplementary Table 1) containing the pxBE3 plasmid. Single colonies were grown and streaked onto a fresh 60-LB plate supplemented with Cm and Spe for selection. The fragment containing edited *phaC* gene was amplified via PCR, utilizing the primers 351-cBE-seq-F/phaC-R-1 and verified by Sanger sequencing.

### 2.7 Shake flask fermentation

A defined minimal medium contained 6 % NaCl, 0.05 % urea, 0.02 % Mg_2_SO_4_, 1.0 % phosphate buffer (containing 14 % K_2_HPO_4_·3H_2_O and 5.2 % KH_2_PO_4_), 1.0 % trace element solution I (comprising 0.5 % Fe (III)–NH_4_-citrate and 0.2 % CaCl_2_·2H_2_O dissolved in 1 M HCl), 0.1 % trace element solution II (comprising 0.01 % ZnSO_4_·7H_2_O, 0.003 % MnCl_2_·4H_2_O, 0.03 % H_3_BO_3_, 0.02 % CoCl_2_·6H_2_O, 0.003 % NaMoO_4_·2H_2_O, 0.002 % NiCl_2_·6H_2_O and 0.001 % CuSO_4_·5H_2_O dissolved in 1 M HCl) and 0.1 % yeast extract was used for shake-flask fermentation. The pH of the medium was adjusted to 8.5−9.0 using 5 M NaOH. Unless otherwise specified, 3 % glucose was added initially as a carbon source. For the production of 3-hydroxypropionic acid (3HP), 1,3-propanediol (Sangon, China) was added at 1 %.

For the first seed preparation, microbial glycerol stock was revived by streaking on fresh 60-LB medium plates. Single colonies were picked from the streaked or newly-conjugated plates and individually inoculated into 2 mL of 60-LB medium for 12–16 h. For the second seed preparation, the strains were transferred into 100 mL shake flasks with 20 mL of fresh 60-LB medium at a volume ratio of 1 % and incubated at 37 °C and 200 rpm for 8 h until the OD_600_ reached 0.6 to 0.8. Subsequently, bacterial seeds were inoculated into a 500 mL shake flask containing 50 mL of defined minimal medium at a ratio of 5 % and incubated at 37 °C for 48 h with 200 rpm shaking. The incubation was then stopped for metabolite production assays.

### 2.8 Analysis of metabolite production

Analysis of metabolite production was conducted using high-performance liquid chromatography (HPLC) on the Agilent 1260 Infinity II LC system (Agilent Technologies, USA). To determine the concentrations of 3HP, the fermentation culture was centrifuged at 10,000×*g* for 10 min at 4 °C. The supernatant was then filtered through a 0.22 μm polyethersulfone (PES) membrane syringe filter (Titan, China). For 3HP analysis, the mobile phase consisted of 5 mM H_2_SO_4_ with a flow rate of 0.5 mL/min, using an Aminex HPX-87H column (300 × 7.8 mm; Bio-Rad, USA). The column temperature was maintained at 55 °C [13]. For each chemical, standard curves were generated using five different concentrations of standard chemicals. The concentrations of desired chemicals in the samples were detected and calculated by comparing them to these standard curves. Approximately 30 mL of fermented bacterial culture was collected in 50 mL centrifuge tubes, weighed, and recorded. Then, the bacterial culture was centrifuged at 10,000×*g* for 10 min at 4 °C. After discarding the supernatant, the centrifuge tubes were sealed with parafilm and the air holes were tied with toothpicks. These tubes were then stored in a refrigerator at −80 °C for 24 h, and quickly transferred to a lyophilizer to eliminate water. The lyophilized organisms and tubes were weighed, and the difference in weights was normalized to determine the cell dry weight (CDW) based on the volume of the bacterial culture. In order to accomplish the esterification process, 30−40 mg of lyophilized cells were added to a 2 mL esterification reagent that contained 0.1 % benzoic acid and 3 % sulfuric acid, both dissolved in absolute methanol. Following this, 2 mL chloroform was added, and the entire reaction was carried out for 4 h at 100 °C. By using gas chromatography with GC-2014 (Shimadzu, Japan), PHB contents were ascertained.

### 2.9 RT-qPCR analysis of relative *ncas9* expression

After being injected for an overnight culture, *H. bluephagenesis* TD1.0 and its newly conjugated strains were transferred by 1 % (v/v) and the cultures were kept going until the OD_600_ reached 0.6 to 0.8 was reached in order to collect the bacteria. The SV Total RNA Isolation System kit (Promega, USA) was used to extract the total RNA from the *H. bluephagenesis* TD strains, and a reverse transcription kit (Thermo, USA) was used to synthesize cDNA. The qPCR was used to detect gene transcription, using iTaq™ Universal SYBR® Green Supermix (Bio-Rad, USA) to prepare reaction sets, with 16S rRNA serving as the internal reference gene. The relative transcription level of the target gene was calculated from the obtained Ct values, and the statistical analysis was conducted.

### 2.10 Statistics

The statistical significance of any variations in metabolite titer between conditions was calculated using analysis of variance (ANOVA) in Graphpad Prism 8. In terms of production experiments, biological replicates for independent cultures were inoculated from distinct *H. bluephagenesis* TD colonies or streaks and cultivated separately.

## 3 Results

### 3.1 Design of CRISPR-BE for *H. bluephagenesis*

**Fig. 1:**
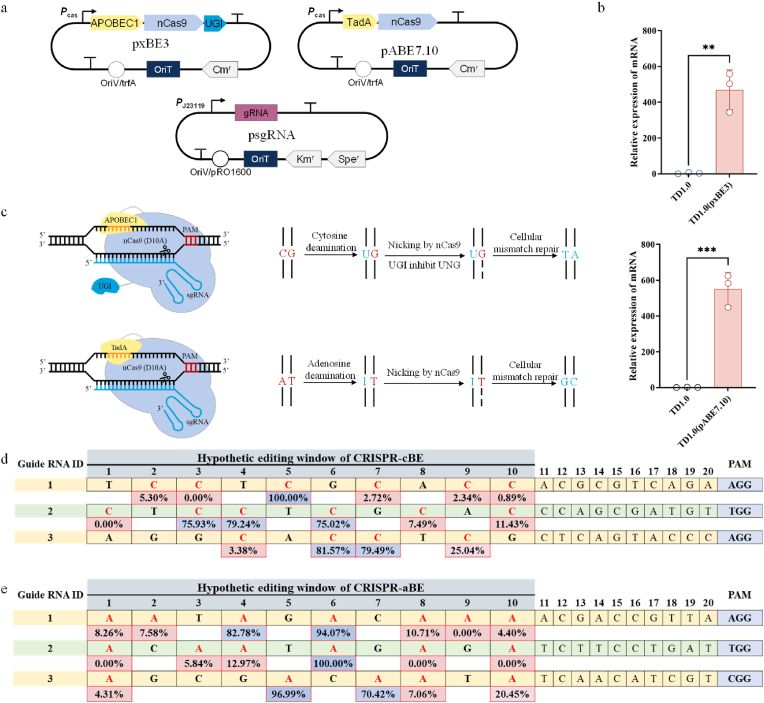
Construction of CRISPR-BE system in *H. bluephagenesis* TD1.0. a. Plasmid configurations of pxBE3, pABE7.10, and psgRNA. The CRISPR-BE plasmid was generated based on pSEVA321 backbone, and the expression of the fusion protein is driven by the promoter *P*_cas_ as indicated. The pxBE3 plasmid was constructed to express a fusion protein of APOBEC1 with Cas9 short cleavage enzyme (nCas9 (D10A)) and UGI, enabling the CRISPR-cBE system to convert cytosine (C) to thymine (T). The plasmid pABE7.10 carried a fusion gene to encode TadA linked to the N terminus of nCas9, which functions in the CRISPR-aBE system to convert adenosine (A) to guanosine (G). The sgRNA sequence is under the control of the promoter *P*_J23119_ by a pRO1600 replicon-based. APOBEC1, rat cytidine deaminase APOBEC1; UGI, uracil glycosylase inhibitor; TadA, the laboratory evolved *E. coli* tRNA-specific adenosine deaminase TadA. **b.** RT-qPCR detection of *ncas9* expression in the strains transformed with pxBE3 (up) or pABE7.10 (down) compared to *H. bluephagenesis* TD1.0. **c.** Schematic representation of RNA-guided cytosine deamination and/or adenine deamination in *H. bluephagenesis* TD1.0. APOBEC1 and TadA were shown in yellow, nCas9(D10A) in blue-purple, sgRNA in light blue, PAM in red. **d–e.** Evaluation of CRISPR-BE targeting selected protospacers from the *H. bluephagenesis* TD1.0 genome. The call secondary peak function of the CLC Main Workbench was used to analyze the mixed trace signal. **d.** The results of CRISPR-cBE. **e.** The results of CRISPR-aBE.

CRISPR/Cas9 genome editing in *H. bluephagenesis* TD requires a homologous arm for each editing target. To address the limitations of CRISPR-Cas9 in systematic metabolic engineering*,* we constructed a DSB-free base editing system in *H. bluephagenesis* TD. This system enables the conversion of targeted cytidine to thymidine or adenosine to guanosine in DNA (Fig. 1a). The two-plasmid system was constructed, for the CRISPR-cBE, pxBE3 for expressing the base editor's enzyme by fusing the rat cytidine deaminase APOBEC1 with a Cas9 nickase (nCas9 (D10A)) and the UGI, and psgRNA for sgRNA transcription. The fusion protein expression is driven by the *P*_cas_ promoter (Fig. 1a). We performed RT-qPCR to detect the success of nCas9 transcription in the strain transformed with pxBE3, named *H. bluephagenesis* TD1.0 (pxBE3). The results showed that the deaminase and nCas9 fusion proteins of the CRISPR-cBE was successfully transcribed (Fig. 1b). The conversion of the targeted C within a C:G base pair to U leads to the formation of a mismatched U:G pair. UGI can inhibit the activity of uracil deoxyribonucleic acid glycosylase (UDG), making it unable to recognize and excise U through base excision repair, thereby preventing reversion to the C:G original base pair [34,35]. The U:G mismatch is also processed by MMR, which prioritizes repairing the nicked strand of a mismatch. The nCas9 nicks the non-edited strand containing the G, which is beneficial for repairing U:G mismatch to the desired T:A (Fig. 1c) [21,36].

Three sgRNA sequences were selected to validate the editing efficiency of CRISPR-cBE in the *H. bluephagenesis* TD1.0 genome. Transfer these sgRNAs into *H. bluephagenesis* TD1.0 (pxBE3), allowed overnight growth for the formation of single colonies, and subsequently scraped off all the colonies using a 60-LB medium to create separate mixed cell pools. CRISPR-cBE's editing efficiency and editing window for each target were determined by conducting Sanger sequencing analysis on the target region of three mixed cell pools. We observed that the conversion of cytidine to thymidine was most efficient within positions 3 to 9. Based on these findings, the hypothetical editing window of CRISPR-cBE was assigned to 7-nucleotide. The editing efficiency for converting cytidines to thymidines within this hypothetic editing window reached 100 % (Fig. 1d).

For CRISPR-aBE, pABE7.10 was constructed by fusing the laboratory evolved *E. coli* tRNA-specific adenosine deaminase TadA (*ec*TadA) with nCas9. The fusion protein expression is driven by the *P*_cas_ promoter, and the deaminase and nCas9 fusion proteins of the CRISPR-cBE was successfully transcribed (Fig. 1b). The conversion of the targeted A within a A:T base pair to I leads to the formation of a mismatched I:T pair. The MMR system, which is responsible for repairing the I:T mismatch, gives priority to repairing the nicked strand. Here, nCas9 specifically cleaves the unedited strand containing T, facilitating the repair of the I:T mismatch to the desired G:C base pair (Fig. 1c). Similar to the validation of CRISPR-cBE, three sgRNA sequences from the *H. bluephagenesis* TD1.0 genome were selected. These sgRNAs were transferred into *H. bluephagenesis* TD1.0 (pABE7.10) to create separate mixed cell pools. We observed that adenosine was more efficiently converted into a guanosine in positions 4 to 10 based on the Sanger sequencing analysis on the target region of three mixed cell pools. Thus, the hypothetical editing window of CRISPR-aBE was assigned to 7-nucleotide. Overall, the editing efficiency for converting adenosines to guanosines within the hypothetical editing window reached 100 % (Fig. 1e).

### 3.2 Editing window and editing efficiency of CRISPR-BE

**Fig. 2:**
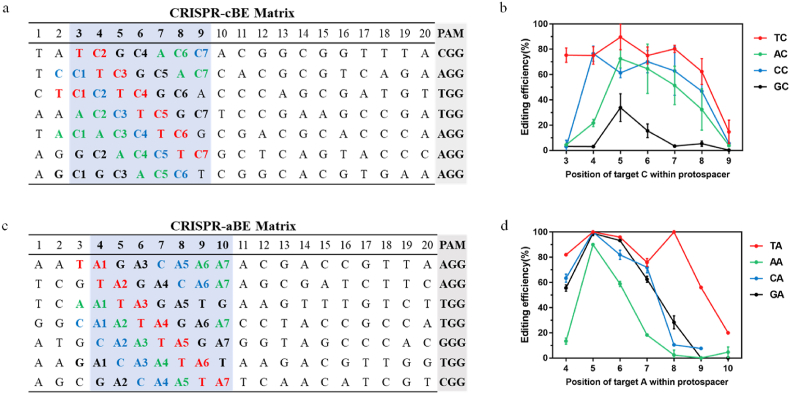
Systematic characterization of CRISPR-BE editing efficiency. CRISPR-BE matrixs presenting 20-nt protospacer containing the target C of NC or target A of NA at different positions, and PAM. **a.** The matrixs of CRISPR-cBE. **b.** The corresponding editing efficiency of CRISPR-cBE. **c.** The matrixs of CRISPR-aBE. **d**. The corresponding editing efficiency of CRISPR-aBE. The editing window was highlighted in blue-purple. Data were shown as mean ± SD of biologically independent conjugation samples (*n* = 3).

Previously reported cytidine deaminase-based base editors typically exhibit an editing window of fewer than 10 nucleotides upstream of the PAM sequence [21,37]. Also, the editing window of the CRISPR-cBE base editor has been previously reported to span 11 to 17 nucleotides upstream of the PAM sequence in *Streptomycetes* species [25]. Notably, significant differences in editing efficiency are observed among different sequence contexts and target nucleotide positions (Fig. 1d and e). A matrix for CRISPR-cBE was designed to systematically evaluate the editing efficiency in *H. bluephagenesis* TD1.0. The distribution of the target C in all seven potential positions was ensured for each NC combination (where N represents T, A, C, or G) (Fig. 2a). Experimental validation was conducted using seven sgRNA identified in nonessential genes (Fig. 2a). These sgRNAs were introduced into *H. bluephagenesis* TD1.0 (pxBE3) to create separate mixed cell pools. The C-to-T conversion efficiency was calculated by Sanger sequencing analysis (Fig. 2b), and the results indicated that CRISPR-cBE could efficiently perform C-to-T editing in positions 3–8 (positions exhibiting an average editing efficiency exceeding 30 % of the average peak-editing efficiency) within the *H. bluephagenesis* TD1.0 genome. Furthermore, the editor exhibited a preference for deamination substrates in the order of TC > CC > AC > GC (Fig. 2b). This finding is consistent with those of other reports [21,25,38]. Positions 5, 6, and 7 achieved a higher editing efficiency and an optimal editing window within the 7-nucleotide editing window was found for CRISPR-cBE (Fig. 2b). These findings lay the groundwork for constructing a metabolic regulatory library based on CRISPR-cBE in *H. bluephagenesis* TD1.0.

Additionally, for the CRISPR-aBE matrix, the target A of each NA combination was distributed in all seven possible positions (Fig. 2c). The seven sgRNAs were transferred into *H. bluephagenesis* TD1.0 (pABE7.10) to create separate mixed cell pools. The A-to-G conversion efficiency was calculated by Sanger sequencing analysis, the results showed that CRISPR-aBE can efficiently perform A to G editing in positions 4–9 in the *H. bluephagenesis* TD1.0 genome (Fig. 2d). Positions 4, 5, 6, and 7 achieved a higher editing efficiency and an optimal editing window within the 7-nucleotide editing window was found for CRISPR-aBE (Fig. 2d).

### 3.3 Applications of CRISPR-cBE in amino acid substitution

**Fig. 3:**
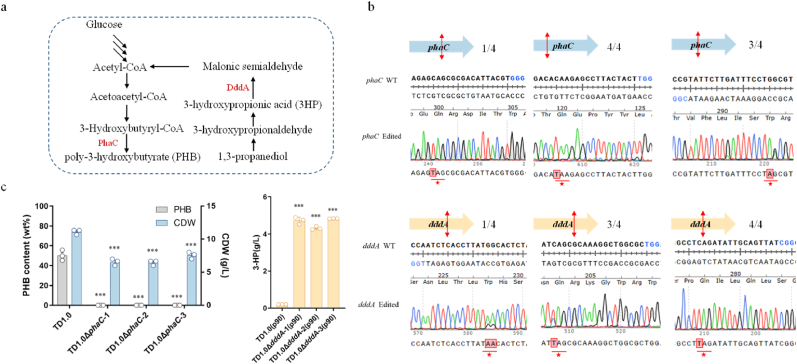
CRISPR-cBE used in *H. bluephagenesis* TD1.0 to deactivate target genes. a. Metabolic pathways for the biosynthesis of 3-hydroxypropionic acid. *phaC*, encoding polyhydroxyalkanoic acid synthase, and *dddA*, encoding 3-hydroxypropionate dehydrogenase, were picked as editing target genes. **b.** Sanger sequencing traces are shown for the region containing the 20-nt protospacer and its PAM. For each gene, three sgRNAs at different positions were selected for knockout, PAM sequences were labeled in blue, and termination codons (TAG, TAA, and TGA) introduced in advance were labeled with red color “★”. Black numbers represent randomly “selected number of successfully introduced stop codon colonies/total number of sequenced colonies”. The red double-headed arrow represents the location where the stop codon was introduced. **c.** Cell dry weight (CDW) and PHB contents were obtained from *H. bluephagenesis* TD1.0 and TD1.0Δ*phaC* by fermentation with a defined minimal medium. 30 mL of fermented bacterial culture was centrifuged after 48 h of incubation at 200 rpm at 37 °C, and the bacterial precipitates were placed at −80 °C overnight and then quickly transferred to a lyophilizer to remove water. *H. bluephagenesis* TD1.0 and TD1.0Δ*dddA* harboring plasmid p90 to produce 3HP, which were grown in a defined minimal medium supplemented with 30 g/L glucose and 10 g/L 1,3-propanediol. Plasmid p90 carries the *aldD*_*Hb*_ gene and the His-tagged *adhP* gene at the C-terminal. *aldD*_*Hb*_, *H. bluephagenesis* aldehyde dehydrogenase; *adhP*, *H. bluephagenesis* alcohol dehydrogenase. Data were shown as mean ± SD of biologically independent samples (*n* = 3). Statistical significance was calculated by ANOVA. *P＜0.05; **P＜0.01; ***P＜0.001.

After careful selection of specific target sites, base editors can induce point mutations and result in either amino acid substitutions or the introduction of stop codons. Two genes, *HPTD01_3821* (encoding PhaC, polyhydroxyalkanoic acid synthase) and *HPTD01_2160* (encoding DddA, 3-hydroxypropionate dehydrogenas), were selected to validate CRISPR-cBE on amino acid replacements in the *H. bluephagenesis* TD1.0 genome (Fig. 3a). Relevant sgRNAs harboring editable cytidines were detected in the target gene region using CRISPy-web [39]. The sgRNAs that can generate a stop codon after mutating the targeted C to T within 25 %–75 % of the target gene sequence were chosen, and the targeted C locating in the optimal editing window was ensured. Three sgRNAs were designed for each of the two genes and separately cloned into CRISPR-cBE plasmid. Subsequently, each of the six sgRNAs was introduced into *H. bluephagenesis* TD1.0 (pxBE3), and the colonies were obtained after overnight growth. A single colony was selected for Sanger sequencing of the targeted region. The results demonstrated successful conversion of the desired target cytidines to thymidines, ultimately leading to the introduction of stop codons (Fig. 3b). Moreover, in all six tested cases, four individual colonies for each case were sequenced to detect strains with target gene inactivation, achieving editing efficiency up to 100 % (Fig. 3b).

In addition, we compare and evaluate the gene inactivation efficiency of CRISPR-deaminase-assisted base editing system with CRISPR/Cas9 system, a gene editing tool commonly used in *H. bluephagenesis* TD. Specifically, the transformation efficiency of sgRNA-donor plasmids into the *H. bluephagenesis* TD (pQ08) strain containing Cas9 was significantly lower than that of sgRNA plasmids into *H. bluephagenesis* TD (pxBE3) strain containing nCas9, differing by more than one order of magnitude (Fig. S1a). Twelve single colonies were randomly selected for colony PCR verification. The results showed that the target gene was not knocked out in all colonies using the CRISPR/Cas9 system (Fig. S1b), while the same target gene were successfully inactivated in all four colonies using the CRISPR-cBE system (Fig. 3b).

After eliminating plasmids of the editing system by continuously subculturing mutant strains more than three times in antibiotic-free medium, the ability of the CRISPR-cBE to redistribute the metabolic flow of *H. bluephagenesis* TD1.0 was assessed by comparing the poly-3-hydroxybutyrate (PHB) contents and 3HP levels in the fermentation broth of the wild-type *H. bluephagenesis* TD1.0, the *H. bluephagenesis* TD1.0Δ*phaC*, and the *H. bluephagenesis* TD1.0Δ*dddA*. The inactivation of the PhaC completely abolished PHB biosynthesis, resulting in a decrease in PHB contents under fermentation conditions (Fig. 3c). The wild type exhibited a distinct white color because of PHB accumulation compared to *H. bluephagenesis* TD1.0Δ*phaC*, indicating that the mutant lost its ability to synthesize PHB (Fig. S2). DddA is a pivotal enzyme in the 3HP degradation pathway of *H. bluephagenesis* TD1.0 (Fig. 3a). After transferring the 3HP production plasmid p90 into *H. bluephagenesis* TD1.0 and *H. bluephagenesis* TD1.0Δ*dddA*, we observed that inactivation of *dddA* gene through the introduction of a stop codon promoted the accumulation of 3HP in the *H. bluephagenesis* TD1.0Δ*dddA* (p90) under fermentation conditions (Fig. 3c). In conclusion, introducing a stop codon at different positions in the target gene could inactivate the corresponding gene function (Fig. 3c).

### 3.4 Simultaneous inactivation of multiple genes

**Fig. 4:**
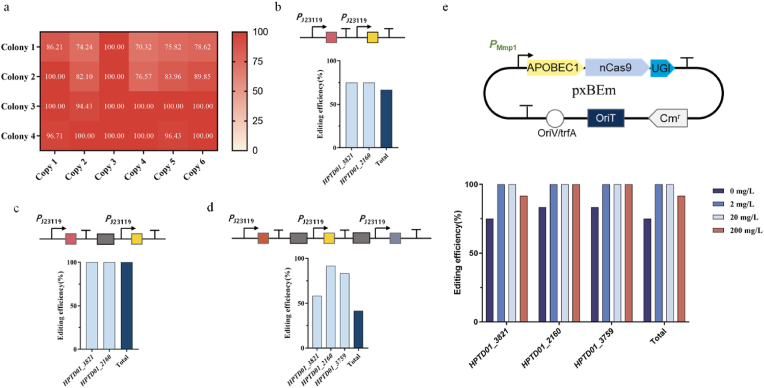
Efficient multiplex-base editing in the *H. bluephagenesis* TD1.0 genome. a. The editing efficiency of the deactivation of the 6-copy IS1086 loci on the genome. Sequencing of four randomly selected colonies verified by PCR using primers from different sites. The secondary peak ratio was obtained using CLC Main Workbench 23.0.5 to calculate the editing efficiency. **b–e.** Editing efficiency of different strategies for deactivation of 2 or 3 genes. The sgRNA used for the *HPTD01_2160* gene was indicated in orange, the sgRNA for *HPTD01_3821* gene was shown in yellow, the sgRNA for *HPTD01_3759* gene was shown in blue-purple and the genes of green fluorescent protein and red fluorescent protein were pointed out in grey. The editing efficiency of twelve randomly selected colonies was calculated as the number of mutants/total number of colonies selected. Total, the efficiency of all genes.

Multiple genes need to be extensively modified concurrently within a biosynthetic pathway because of the intricate regulation of cellular metabolism in organisms. Simultaneous gene inactivation during a single round of editing can significantly reduce the workload and time required for metabolic regulation processes. CRISPR-cBE facilitates efficient multiplex-base editing in genomes through the design of sgRNA arrays. To demonstrate the potential of the multiple loci editing, we replaced sgRNA with the sgRNA_IS1086, targeting six-copy IS1086 loci on the genome. Editing events were observed at all six IS1086 loci in four randomly picked colonies (Fig. 4a). The editing efficiency was all over 70 % and many of them reached 100 %, underscoring the versatility and efficiency of CRISPR-cBE in genome engineering.

Given the positive results above, we further explored the potential of CRISPR-cBE in targeting multiple different loci on the genome. PCR-based gene synthesis was applied to obtain a single DNA construct for multiplexing sgRNAs [40]. The editing efficiency was verified via inactivation of two and three endogenous genes. In the case of inactivating two genes, the editing region of twelve strains were amplified and sequenced for each target. The results demonstrated that the editing efficiency was relatively low when two sgRNAs adjacent to each other, and eight out of twelve randomly selected strains (66.67 %) exhibited successfully edited in both genes (Fig. 4b). Interestingly, when the sgRNAs were separated by a green fluorescent protein, all twelve randomly selected strains presented both edited genes, resulting in an editing efficiency of 100 % (Fig. 4c). Furthermore, regulating two sgRNAs in opposite promoter orientations exhibited a lower editing efficiency compared to regulating sgRNAs in the same promoter orientation (Fig. S3). For the inactivation of three genes, the editing region of the twelve strains that were amplified and sequenced for each target, five strains (41.67 %) showed successfully edited all three genes, while six other strains showed edited only two genes and one strain showed edited only one gene (Fig. 4d). In the meanwhile, the editing efficiency of three sgRNAs expression module adjacent to each other was lower than three sgRNAs separated with green and red fluorescent proteins (Fig. S4).

To further enhance the inactivation efficiency of multiple genes, we optimized CRISPR-cBE system by altering the expression pattern of sgRNA arrays and regulating the protein level of the deaminase and nCas9 fusion protein. However, no successfully edited colonies were detected when multiple sgRNAs were expressed as a polycistronic cluster. Therefore, it is preferable to transcribe each sgRNA cassette as a single gene unit rather than transcribing multiple sgRNAs as a cluster in *H. bluephagenesis* TD. It was reported that adding a degradation tag (LVA tag) to the C-terminus of the fusion protein can reduce its toxicity, thereby improving the efficiency of base editing [22]. However, after adding the LVA tag, the base editing efficiency decreased significantly in *H. bluephagenesis* TD, possibly due to the degradation tag reducing the level of the fusion protein. Therefore, increasing the protein level of the fusion protein may enhance the editing efficiency. By substituting the *P*_cas_ promoter with the inducible promoter *P*_Mmp1_, we ultimately achieved optimized CRISPR-cBE system and 100 % editing efficiency in inactivating three genes (Fig. 4e). These findings demonstrate that our system successfully facilitates the simultaneous editing of multiple bases with high efficiency. In summary, our developed multiplexed base editing system demonstrates a rapid and efficient approach to obtain recombinant *H. bluephagenesis* TD strains with multiple inactivated genes.

## 4 Discussion

*H. bluephagenesis* TD and its recombinant strains have been demonstrated as economically viable platforms for the production of bulk chemical [4]. However, the limited methods and tools for manipulating and metabolic engineering severely hinder the full potential of *H. bluephagenesis* TD as a chassis. In order to effectively regulate the complexity of natural metabolic pathways for desired products, a systematic metabolic engineering approach is imperative to achieve efficient biosynthesis of target products through intensively engineered biosynthetic pathways. In this study, we implemented highly efficient CRISPR/nCas9 guide base editors for edit multiple loci in *H. bluephagenesis* TD1.0, including C-to-T (CRISPR-cBE) and A-to-G (CRISPR-aBE) base editors. Without the need for a homologous arm and the introduction of DSBs, CRISPR-BE can be used for efficiently editing target genes with single-base pair resolution in *H. bluephagenesis* TD1.0.

The introduction of a single-strand DNA nick in the nonedited DNA strand by nCas9 can enhance the effectiveness of MMR, leading to an increased frequency of base editing [21]. We used nCas9 as the deaminase delivery system in our CRISPR-BE system (Fig. 1a). Our findings are consistent with previous studies [21,25], revealing that TC is the favored editing substrate for CRISPR-cBE (Fig. 2b), while TA is the favored substrate for CRISPR-aBE (Fig. 2d). To validate CRISPR-cBE-mediated amino acid substitutions in the *H. bluephagenesis* TD 1.0 genome, we constructed a null mutant through stop codon introduction. For single gene inactivation, we achieved an editing efficiency of 100 % (Fig. 3b). Remarkably, when inactivating two genes, all twelve strains were successfully edited with an editing efficiency of 100 % (Fig. 4c). For the inactivation of three genes simultaneously, we successfully edited all three genes in five strains out of the twelve, achieving an editing efficiency of 41.67 % (Fig. 4d). By substituting the *P*_cas_ promoter with the inducible promoter *P*_Mmp1_, we optimized CRISPR-cBE system and ultimately achieved 100 % editing efficiency in inactivating three genes (Fig. 4e).

In summary, our study demonstrates that CRISPR-BE can accomplish genome editing with exceptional efficiency at a precise single-base pair level, without the need for a DSB. CRISPR-BE holds immense potential for future protein evolution through in situ substitution of crucial residues, identification of gene function via editing libraries, and engineering entire pathways using multiplexed sgRNAs within a single construct. Overall, the utilization of the CRISPR-BE system for base editing represents a highly efficient approach for accelerating the establishment of *H. bluephagenesis* TD cell factories, with potentially widespread applications.

## Data availability

Data supporting the findings of this work are available within the paper and its supplementary information files. All relevant data are available from the authors upon reasonable request.

## CRediT authorship contribution statement

Yulin Zhang: Conceptualization, Methodology, Formal analysis, Data curation, Investigation, Writing – original draft, Writing – review & editing, Visualization. Yang Zheng: Investigation, Data curation, Validation. Qiwen Hu: Investigation, Formal analysis, Writing – review & editing. Zhen Hu: Methodology, Resources, Writing – review & editing. Jiyuan Sun: Methodology. Ping Cheng: Investigation, Resources. Xiancai Rao: Writing – original draft, Conceptualization, Supervision. Xiao-Ran Jiang: Writing – original draft, Conceptualization, Supervision, Funding acquisition. All authors have read and agreed to the published version of the manuscript.

## Declaration of competing interest

The authors declare that they have no known competing financial interests or personal relationships that could have appeared to influence the work reported in this paper.
