# Supplementary material for: Simultaneous multiplex genome loci editing of Halomonas bluephagenesis using an engineered CRISPR-guided base editor
Source: Synth Syst Biotechnol. 2024 Apr 25;9(3):586–93. doi: 10.1016/j.synbio.2024.04.016 (PMC11076302; doi:10.1016/j.synbio.2024.04.016)
Supplement: Multimedia component 1 [file mmc1.docx]

**Supplementary data for**

**Simultaneous Multiplex Genome Loci Editing of *Halomonas bluephagenesis* using an Engineered CRISPR-guided base editor**

**Figure S1**. **Efficiency comparison of the CRISPR/Cas9 and CRISPR-cBE systems.**

The sgRNA_phaC2 was chosen to modify the *phaC* gene. 20-bp sgRNA sequence was knocked out by CRISPR/Cas9, and stop codon was introduced by CRISPR-cBE. **a.** Transformation efficiency of CRISPR/Cas9 and CRISPR-cBE systems. The number of colonies was counted by Icount 50 automatic colony counter (Hangzhou Shineso Technology Co., Ltd.). Colony Forming Units (CFU) = (The number of colonies) × (dilution multiple). **b.** Electropherogram used to validate the sgRNA_phaC2 sequence knockout by CRISPR/Cas9. Internal primer colony PCR was performed on twelve randomly chosen single colonies. The theoretical amplification fragment size of wild type was 1877 bp, while The knockout strain should theoretically not be amplified with any bands. M: DNA Marker; WT: wild type; NC: negative control.

**
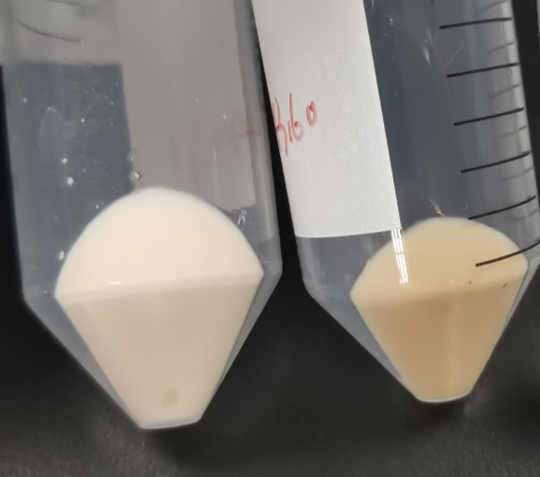
**

**Figure S2**. **Bacterial precipitates of *H. bluephagenesis*** **TD1.0 and TD1.0Δ*phaC* after fermentation.**

Bacterial pellets of 30 mL of TD1.0 culture (left), and TD1.0Δ*phaC* fermented culture (right).

**Figure S3**. **Editing efficiency of** **two sgRNAs in opposite promoter orientations.**

12 colonies were cultured and each gene in the genome was sequenced. Editing efficiency was calculated as No. of mutants/Total colonies selected. Total, the efficiency of all genes.

**Figure S4**. **The editing efficiency of three sgRNAs expression module adjacent to each other.**

12 colonies were cultured and each gene in the genome was sequenced. Editing efficiency was calculated as No. of mutants/Total colonies selected. Total, the efficiency of all genes.

**Supplementary** **Table 1** | Strains used in this study

| **Strains** | **Description** | **Sources** |
| --- | --- | --- |
| *H. bluephagenesis* TD1.0 | A novel T7-like RNA polymerase-integrated derivative of the wild-type *H. bluephagenesis* strain TD01 | [1] |
| *E. coli* S17-1 | Conjunction donor, *tra* genes of RP4 plasmid integrated on the chromosome; recA, proA, thi-1, endA1 | [2] |
| TD1.0 (pQ08) | *H. bluephagenesis* TD1.0 carrying pQ08 plasmid | This study |
| TD1.0 (pxBE3) | *H. bluephagenesis* TD1.0 carrying pxBE3 plasmid | This study |
| TD1.0 (pABE7.10) | *H. bluephagenesis* TD1.0 carrying pABE7.10 plasmid | This study |
| TD1.0 (pxBEm) | *H. bluephagenesis* TD1.0 carrying pxBEm plasmid | This study |

**Supplementary** **Table 2** | Plasmids used in this study

| **Plasmids** | **Description** | **Sources** |
| --- | --- | --- |
| pSEVA321 | An expression vector, trfA replication origin, *oriT*, Cm^r^ | [3] |
| pSEVA341 | pRO1600/ColE1 replication origin, oriT, Kan^r^ and Spe^r^ | [3] |
| pQ08 | pSEVA321 derivative, *S. pyogenes* cas9, Cm^r^ | [4] |
| p90 | pSEVA321 derivative, *P*_Re_-synthetic stronger RBS-*aldD_Hb_*-*adhP*-His-tag, Kan^r^ and Spe^r^ | [5] |
| pxBE3 | pSEVA321 derivative, *P*_cas_-APOBEC1-nCas9-UGI, Cm^r^ | This study |
| pABE7.10 | pSEVA321 derivative, *P*_cas_-TadA-nCas9-UGI, Cm^r^ | This study |
| psgRNA | pSEVA341 derivative, *P*_J23119_-sgRNA, Kan^r^ and Spe^r^ | This study |
| pxBEm | pSEVA321 derivative, *P*_Mmp1_-APOBEC1-nCas9-UGI, Cm^r^ | This study |

**Supplementary** **Table 3** | Primers used in this study

| **Name** | **Sequence (5'-3')** | **Description** | **Target band** |
| --- | --- | --- | --- |
| 264-1 | TTTTATTTTAGGAGGCAAAAATGTCCTCCGAGACCGGC | For the construction of plasmid pxBE3 | 775 bp |
| 264-2 | CCTATTGAGTATTTCTTATCGGACTCCGGGGTGGCGGAC |  |  |
| 264-3 | GATAAGAAATACTCAATAGGCTTAGCGATCGGCACAAATAGCGTCG |  | 4101 bp |
| 264-4 | GTCACCTCCTAGCTGACTCAAATC |  |  |
| 264-5 | TGAGTCAGCTAGGAGGTGACTCCGGCGGCTCCACCAAC |  | 304 bp |
| 264-6 | ATCTTCATCTAAAATATACTTCACAGCATCTTGATCTTGTTCTCG |  |  |
| 264-7 | AGTATATTTTAGATGAAGATTATTTCTTAATCTAGACATGAGC |  | 4304 bp |
| 264-8 | TTTTGCCTCCTAAAATAAAAAGTTTAAATTAAATCCATAATGAG |  |  |
| BsaI-J23119-R | AAAGGTCTCACTAGTATTATACCTAGGACTGAGC | For the construction of plasmid psgRNA | 6662 bp |
| BsaI-sgRNA-F | AAAGGTCTCGAGCTAGAAATAGCAAGTTAAAATAAGG |  |  |
| 364-1 | TTTTATTTTAGGAGGCAAAAATGAGCGAGGTCGAGTTCTCC | For the construction of plasmid pABE7.10 | 1231 bp |
| 364-2 | CCTATTGAGTATTTCTTATCCGAGCCGCCGCTGCTGCC |  |  |
| cMartix1-seq-F | CTCCAGACTCCCTATGGGCG | For amplification of sequenced fragments containing CRISPR-cBE Matrix protospacers |  |
| cMartix1-seq-R | ACATCTGGTAATGCGGCCAC |  |  |
| cMartix2-seq-F | CAGCACGCTGACTGACATAC |  |  |
| cMartix2-seq-R | GCAGCGCAGCATGTTATACT |  |  |
| cMartix3-seq-F | ACCACTTCCGCACCATGAGC |  |  |
| cMartix3-seq-R | GTTTAGCGTGTTGCCGTGGC |  |  |
| cMartix4-seq-F | CGCCCAAGCCGGAGGTAATC |  |  |
| cMartix4-seq-R | CGCCCTAAATGATCACAGCC |  |  |
| cMartix5-seq-F | CCACTACCCCATTGCACACC |  |  |
| cMartix5-seq-R | GATCTGCATAACGCTGAGCG |  |  |
| cMartix6-seq-F | GGCGGCATTGATCCTTATGC |  |  |
| cMartix6-seq-R | CAAGTAACGTACCGGGTGCC |  |  |
| cMartix7-seq-F | CAGGGCTTAATCAGGCGCTG |  |  |
| cMartix7-seq-R | CGCTCCTTAGCCACCGAGGC |  |  |
| cMartix8-seq-F | TGGTGGCCAGTACCACTCTC |  |  |
| cMartix8-seq-R | CCTTAAGTAGGCGCAGTCGC |  |  |
| cMartix9-seq-F | AGGCGTCGATTCGACGCTTG |  |  |
| cMartix9-seq-R | GATCTCGGATTGCGTGTGCC |  |  |
| cMartix10-seq-F | GGCAATATAGGCCCGCCCTA |  |  |
| cMartix10-seq-R | CTGGCCAGCAAGTCACCGTG |  |  |
| cMartix11-seq-F | GCCCGGCATCGAAGATCACG |  |  |
| cMartix11-seq-R | GGCGCGAGGTTACTCGGACG |  |  |
| cMartix12-seq-F | CCAGCAGCAGTAACGAGAGC |  |  |
| cMartix12-seq-R | ATGATGATGGTCATGTGGCC |  |  |
| cMartix13-seq-F | CCTACGGTCAACACTACGCC |  |  |
| cMartix13-seq-R | TGAGATCCCGGATGCGATCG |  |  |
| cMartix14-seq-F | ATTGAGCAGCACCGTCATCC |  |  |
| cMartix14-seq-R | GCAGAAGGCATTGGCGTTGG |  |  |
| aMartix1-seq-F | GCCCAATTGTGAACAGCACA | For amplification of sequenced fragments containing CRISPR-aBE Matrix protospacers |  |
| aMartix1-seq-R | TAGCGGTAACGGTCATTGGC |  |  |
| aMartix2-seq-F | TTGTGTAGCAGGCGCTTTTT |  |  |
| aMartix2-seq-R | AATCGCGACCAAGACCTTTC |  |  |
| aMartix3-seq-F | GTGCGTCTGGATATTCGCCG |  |  |
| aMartix3-seq-R | GAGTGTCCAGACAAGCAGCA |  |  |
| aMartix4-seq-F | GTGAGTCCTACCGTCGTCGG |  |  |
| aMartix4-seq-R | CTGCGTTCAGCTTCGCTATG |  |  |
| aMartix5-seq-F | CTGTGGTGGAGGCATCGATT |  |  |
| aMartix5-seq-R | GTGCAGCTGATAAGGCTTCC |  |  |
| aMartix6-seq-F | CGACGATATACCTCAACGGC |  |  |
| aMartix6-seq-R | AGCAACTACAACAGTTCGCC |  |  |
| aMartix7-seq-F | TGGATAATGGCTTGGGCTGC |  |  |
| aMartix7-seq-R | GGCTGCGCTATCAATAGACG |  |  |
| aMartix8-seq-F | TTAGCCTGACGTCTCAGCTG |  |  |
| aMartix8-seq-R | GGCACGATAACGACGGTTAC |  |  |
| aMartix9-seq-F | CAAACGTCGCCAGAGTTTCA |  |  |
| aMartix9-seq-R | CCGTCTGCATCTTGATGGCG |  |  |
| aMartix10-seq-F | TGCCGATTCCGGTACCCATG |  |  |
| aMartix10-seq-R | TCTCCAGTCAGTTCAGCCCA |  |  |
| aMartix11-seq-F | TCGCAGAGTCACCTTGGTTT |  |  |
| aMartix11-seq-R | GCCGTCATGCAGATTCGATT |  |  |
| aMartix12-seq-F | CCGCTACGCAATGCATCTGA |  |  |
| aMartix12-seq-R | GCGTAACCATGTTAGCCAGC |  |  |
| aMartix13-seq-F | ATACGGCGTTACTTCGCCAC |  |  |
| aMartix13-seq-R | CGAGTGGTGTATCAGCGCTT |  |  |
| aMartix14-seq-F | CCTACGCCCGATGCTACCTA |  |  |
| aMartix14-seq-R | GCGATGGGCATGGGTATTGT |  |  |
| 351-cBE-seq-F | GTACGCATGACCGATCGAAG | For *H. bluephagenesis* genomic sites PCR amplification and sequencing |  |
| phaC-R-1 | TAGTAGCGGCCCATCGTGGG |  |  |
| dddA-inner-F | AATCGACTCAGTGCCAATCC |  |  |
| dddA-inner-R | GGATGGCGCCATGCTCATCG |  |  |
| 3759-F | TCCGCTTACTGCTCCTGGTG |  |  |
| 3759-R | TTGTGCAGCTGCTACGCATT |  |  |
| IS1086-1 | GCCTCAAGAACTCGGTAGAC |  |  |
| IS1086-2 | GGAGGCCCGTATTCACAGTC |  |  |
| IS1086-3 | ACGGAGGGGATAACGTATCC |  |  |
| IS1086-4 | TAAATCGCCTACACGCTCAG |  |  |
| IS1086-5 | TCAATGGCTAGCTCACCACC |  |  |
| IS1086-6 | CCATGCTAGTGCCCAGTGCG |  |  |
| IS1086-7 | CGTAATGAAGTTATCCATAAGGGC |  |  |
| IS1086-8 | GCCTCAGCATAGATAGAGTCATCAC |  |  |
| IS1086-9 | AATATGCCCGCGAGCGTCAC |  |  |
| IS1086-10 | GCGCCTGAACATCTAACCCG |  |  |
| IS1086-11 | ACTGACCGTACCCGGATATC |  |  |
| IS1086-12 | CTCTCCCGCACTGAGGTTCG |  |  |
| Cas9-qPCR-F | GGGGAAACTGGAGAAATTGTCTGGG | For RT-qPCR amplification |  |
| Cas9-qPCR-R | GGAGAATCCGCCTGTCTGTACT |  |  |
| 16S-qPCR-F | ATCGGGAGGAATACCAGTG |  |  |
| 16S-qPCR-R | CGTTTACGGCGTGGACTA |  |  |

**Supplementary Table 4** | The sgRNA sequences used in this study

| **Names** | **Target gene** | **Sequence (5’ to 3’)** | **PAM** |
| --- | --- | --- | --- |
| sgRNA_phaC1 | *phaC* | AGAGCAGCGCGACATTACGT | GGG |
| sgRNA_phaC2 | *phaC* | GACACAAGAGCCTTACTACT | TGG |
| sgRNA_phaC3 | *phaC* | ACGCCAGGAAATCAAGAATA | CGG |
| sgRNA_dddA1 | *dddA* | AGAGTGCCATAAGGTGAGAT | TGG |
| sgRNA_dddA2 | *dddA* | ATCAGCGCAAAGGCTGGCGC | TGG |
| sgRNA_dddA3 | *dddA* | GCCTCAGATATTGCAGTTAT | CGG |
| sgRNA_IS1086 | IS1086 | CTGCCGAGAGACCATCTACA | CGG |
| sgRNA_1 | *HPTD01_3759* | TCTGCCCAACGCGCCATACT | CGG |

**Supplementary Table 5** | The protospacers used in this study

| **Names** | **Protospacer sequence (5’ to 3’)** | **PAM** |
| --- | --- | --- |
| cBE_Matrix_sgRNA_1 | TATCGCACCACGGCGGTTTA | CGG |
| cBE_Matrix_sgRNA_2 | ACTCGCACCACTGCACAACC | CGG |
| cBE_Matrix_sgRNA_3 | ACCTCGCACCGTGCGGGCCT | GGG |
| cBE_Matrix_sgRNA_4 | TCCTCGCACCACGCGTCAGA | AGG |
| cBE_Matrix_sgRNA_5 | CTCCTCGCACCCGGACGCCG | TGG |
| cBE_Matrix_sgRNA_6 | CTCCTCGCACCCAGCGATGT | TGG |
| cBE_Matrix_sgRNA_7 | GCACCTCGCTCAATGCCTGC | GGG |
| cBE_Matrix_sgRNA_8 | AAACCTCGCTCCGAAGCCGA | TGG |
| cBE_Matrix_sgRNA_9 | CACACCTCGCCGCTCGACCT | CGG |
| cBE_Matrix_sgRNA_10 | TACACCTCGCGACGCACCCA | AGG |
| cBE_Matrix_sgRNA_11 | CAGCACCTCGCAGTTGCTGT | TGG |
| cBE_Matrix_sgRNA_12 | AGGCACCTCGCTCAGTACCC | AGG |
| cBE_Matrix_sgRNA_13 | TGCGCACCTCTTCGCGCAAC | TGG |
| cBE_Matrix_sgRNA_14 | AGCGCACCTCGGCACGTGAA | AGG |
| aBE_Matrix_sgRNA_1 | AATAGACAAAACGACCGTTA | AGG |
| aBE_Matrix_sgRNA_2 | CGTAGACAAGTTCCTGAGGC | AGG |
| aBE_Matrix_sgRNA_3 | TGGTAGACAAAGCGGGCGCG | TGG |
| aBE_Matrix_sgRNA_4 | TCGTAGACAAAGCGATCTTC | AGG |
| aBE_Matrix_sgRNA_5 | ACAATAGAGATCTTCCTGAT | TGG |
| aBE_Matrix_sgRNA_6 | TCAATAGATGAAGTTTGTCT | TGG |
| aBE_Matrix_sgRNA_7 | GGCAATAGAACCTACCGCCA | TGG |
| aBE_Matrix_sgRNA_8 | CGCAATAGAAATACGCGCAC | CGG |
| aBE_Matrix_sgRNA_9 | TGTCAATAGACGTCGTAAAG | GGG |
| aBE_Matrix_sgRNA_10 | ATGCAATAGAGGTAGCCCAC | GGG |
| aBE_Matrix_sgRNA_11 | AGCGACAATATCAACATCGT | CGG |
| aBE_Matrix_sgRNA_12 | AAGACAATATAAGACGTTGG | TGG |
| aBE_Matrix_sgRNA_13 | CCGACAATATAAAGCTAGCA | AGG |
| aBE_Matrix_sgRNA_14 | TGAGACAATATGACGACTGG | TGG |

**Reference**

1. Zhao H., et al. Novel T7-like expression systems used for *Halomonas.* Metab Eng, 2017;39:128-140*.*

2. Simon R. High frequency mobilization of gram-negative bacterial replicons by the in *vitro* constructed Tn5-Mob transposon*.* Mol Gen Genet, 1984;196;3:413-420*.*

3. Silva-Rocha R., et al. The Standard European Vector Architecture (SEVA): a coherent platform for the analysis and deployment of complex prokaryotic phenotypes*.* Nucleic Acids Res, 2013;41:D666−675*.*

4. Qin Q., et al. CRISPR/Cas9 editing genome of extremophile *Halomonas spp.* Metab Eng, 2018;47:219−229*.*

5. Jiang X. R., et al. Hyperproduction of 3-hydroxypropionate by *Halomonas bluephagenesis.* Nat Commun, 2021;12;1:1513*.*
